# TgKDAC4: A Unique Deacetylase of *Toxoplasma*’*s* Apicoplast

**DOI:** 10.3390/microorganisms11061558

**Published:** 2023-06-12

**Authors:** Mariana Sayuri Ishikawa Fragoso, Caroline Moraes de Siqueira, Francisca Nathália Luna Vitorino, Alexandre Zanatta Vieira, Érica Santos Martins-Duarte, Helisson Faoro, Júlia Pinheiro Chagas da Cunha, Andréa Rodrigues Ávila, Sheila Cristina Nardelli

**Affiliations:** 1 Instituto Carlos Chagas, Fundação Oswaldo Cruz, Curitiba 81350-010, Brazil; 2 Special Laboratory of Cell Cycle, Center of Toxins, Immune Response and Cell Signalling (CeTICS), Instituto Butantan, São Paulo 05503-900, Brazil; 3 Department of Parasitology, Instituto de Ciências Biológicas, Universidade Federal de Minas Gerais, Belo Horizonte 31270-901, Brazil

**Keywords:** Desacetilase, *Toxoplasma*, apicoplast

## Abstract

*Toxoplasma gondii* is an obligate intracellular parasite of the phylum Apicomplexa and causes toxoplasmosis infections, a disease that affects a quarter of the world’s population and has no effective cure. Epigenetic regulation is one of the mechanisms controlling gene expression and plays an essential role in all organisms. Lysine deacetylases (KDACs) act as epigenetic regulators affecting gene silencing in many eukaryotes. Here, we focus on TgKDAC4, an enzyme unique to apicomplexan parasites, and a class IV KDAC, the least-studied class of deacetylases so far. This enzyme shares only a portion of the specific KDAC domain with other organisms. Phylogenetic analysis from the TgKDAC4 domain shows a putative prokaryotic origin. Surprisingly, TgKDAC4 is located in the apicoplast, making it the only KDAC found in this organelle to date. Transmission electron microscopy assays confirmed the presence of TgKDAC4 in the periphery of the apicoplast. We identified possible targets or/and partners of TgKDAC4 by immunoprecipitation assays followed by mass spectrometry analysis, including TgCPN60 and TgGAPDH2, both located at the apicoplast and containing acetylation sites. Understanding how the protein works could provide new insights into the metabolism of the apicoplast, an essential organelle for parasite survival.

## 1. Introduction

Post-translational modifications (PTMs) are essential to modulate several cellular mechanisms. Acetylation is one of the most studied [1], first identified as a PTM on histones [2]. The acetyl group can also be added to other proteins from different cellular compartments, triggering a variety of molecular mechanisms [3]. Such PTMs are reversible since the acetyl group added to a lysine amino acid by lysine acetyltransferases (KATs) can be removed by lysines deacetylases (KDACs) [4,5,6]. Acetylation sites can be identified by a complete screening of acetylated proteins using mass spectrometry, leading to the identification of a cell’s acetylome. The acetylome of several prokaryotes and eukaryotes has already been revealed [7,8,9]. In 2012, Jeffers and Sullivan analyzed the acetylome of the protozoan *Toxoplasma gondii*, an obligate intracellular parasite from the phylum Apicomplexa and the cause of toxoplasmosis [10]. Toxoplasmosis is a worldwide disease without treatment in the chronic phase [11]. Interestingly, acetylation is associated with some essential functions of *T. gondii*, such as the virulence of certain strains and cellular differentiation during its life cycle [12,13]. *T. gondii* has a complex life cycle, alternating between two hosts: felines, which are the definitive hosts where the sexual phase occurs, and other warm-blooded animals, including humans, which are intermediate hosts where the asexual phase occurs. During the asexual phase, two forms of *T. gondii* can be distinguished, tachyzoites, which are the proliferative form responsible for the acute phase of the disease, and bradyzoites, which form cysts that can remain latent for the rest of the host’s life and are therefore found in the chronic phase of toxoplasmosis [14]. The differentiation between these forms occurs as a means of evading the host’s immune system, and is tightly regulated by controlling gene expression [15]. There are significant differences in the protein profile during *Toxoplasma* differentiation, and since post-translational modifications are rapid and dynamic, they may possibly be one of the main forms of regulation during the transition between these forms [16,17,18]. The genes of some KATs and KDACs have already been identified in the genome of *T. gondii*. The KAT enzymes are TgGCN5-A, TgGCN5-B, TgMYST-A, and TgMYST-B, while the KDACs are KDAC 1-5, Sir2A, and Sir2B [19,20]. KDACs are usually involved in histone deacetylation, often leading to gene silencing. In addition, these enzymes have a versatile function and modify a variety of substrates. For instance, KDACs can also modulate enzymatic activity, protein localization, protein stabilization, and molecule interactions [5,21]. Hence, KDACs act in an array of cellular processes, including apoptosis, the cell cycle, DNA repair, and cytoskeleton organization [5,22].

Phylogenetic analyses group eukaryotic KDACs into four classes with specific features [23]. Classes I, II, and IV are considered classical and are made up of enzymes that use zinc as a cofactor. KDACs from classes I and II are homologous to the enzymes Rpd3 and HDA1 of *Saccharomyces cerevisiae*, respectively. Class IV is classified separately, as it has no similarity with either Rpd3 or Hda1. Human HDAC11 is the most widely characterized enzyme of class IV. Class III KDACs are considered non-classical and consist of enzymes that use NAD+ as a cofactor [20,23,24,25].

Despite the classification of the KDACs of *T. gondii* into the above classes, TgKDAC4 has not been assigned to any particular class [20]. Moreover, although different KDAC genes have been identified in the *T. gondii* genome, only the function of TgKDAC3 has been studied as it relates to parasite differentiation [12,19,26]. TgKDAC3 binds to promoters of bradyzoite genes that are not transcribed in tachyzoites, while TgGCN5-A binds to promoters of transcriptionally active genes, demonstrating the importance of these enzymes for the regulation of gene expression during the differentiation of *T. gondii* [12].

Due to the important role of protein acetylation on the function of eukaryotic cells, KATs and KDACs have been explored as targets for the treatment of several diseases, with inhibitors of these enzymes of particular interest for treating tumors [27,28,29]. KDAC inhibitors have also shown potential for treating several parasitic diseases, including toxoplasmosis. Low concentrations of TST and SAHA, two inhibitors initially identified for cancer treatment, also inhibited the in vitro proliferation of three genotypes of *T. gondii* [30]. FR235222, an inhibitor of *T. gondii* KDAC3, also significantly decreased parasite growth and induced the in vitro differentiation of tachyzoites into bradyzoites [26].

Molecules of Acetyl-CoA are the source of the acetyl group added by KATs to proteins. In *Toxoplasma*, there are four enzymes capable of generating Acetyl-CoA, one of which is located in the apicoplast, a unique organelle of Apicomplexa [31,32]. The apicoplast is an organelle of endosymbiotic origin acquired through secondary endosymbiosis, a process that involves the engulfment of a photosynthetic red alga by the apicomplexan ancestor. As a result, the apicoplast has four membranes, of which two inner membranes originated from cyanobacteria, one from algae, and the other from the host’s endosomal compartment [33,34,35,36]. Due to this cyanobacterial origin, the proteins involved in the maintenance of its genome are of bacterial origin [37,38,39].

The *Toxoplasma* apicoplast supports essential pathways for the parasite’s survival, such as the synthesis of type II fatty acids, isoprenoids, and heme [35,40,41,42]. Acetylated proteins were identified in the apicoplast [10], but the full repertoire of enzymes associated with these acetylations and deacetylations is still unknown.

To better understand the process of proteins deacetylation in *Toxoplasma*, we aim to characterize the TgKDAC4, a class IV KDAC with peculiar characteristics. Besides belonging to the least understood class, TgKDAC4 is similar to bacterial deacetylases and may demonstrate an unusual function. We describe a phylum-specific KDAC with an unexpected cellular location and propose the association of this enzyme with the deacetylation of essential proteins to metabolic pathways of the parasite.

## 2. Materials and Methods

### 2.1. Parasites Culture

Tachyzoytes of RH*∆hxgprt∆ku80* and the mutant tag line (TgKDAC4-HA) were cultured in confluent NHDF (Normal Human Dermal Fibroblasts-Lonza, Basel, Switzerland) in Dulbecco’s modified Eagle medium (DMEM-GIBCO, Stanley Road, Grand Island, NY, USA) supplemented with 10% FBS, 2 mM L-glutamine, 100 U/mL penicillin, and 100 mg/mL streptomycin at 37 °C under a 5% CO_2_ atmosphere.

### 2.2. Transfection and Endogenous Tagging Confirmation

Initially, to obtain the complete sequence of TgKDAC4, we extracted RNA using the RNeasy Mini Kit (QIAGEN, Hilden, Germany), following the manufacturer’s instructions. Messenger RNAs were amplified from the extracted RNA using oligodT. The obtained cDNA was used as a template for a conventional Polymerase Chain Reaction (PCR) for regions of TgKDAC4, and then subjected to sequencing to establish the entire open reading frame. One 1000-bp fragment corresponding to the C-terminal region of *tgkdac4*, without a termination codon, was amplified by PCR and cloned into the plasmid pLIC.HA.HXGPRT using a ligation-independent cloning (LIC) approach, as previously described by Huynh and Carruthers [43]. The sequences of oligonucleotides used were as follows (LIC sequences in bold): TgKDAC4 F, **TACTTCCAATCCAATTTAATGC**TACGCTCATGGCCTAGGC; TgKDAC4 R, **TCCTCCACTTCCAATTTTAGC**CTCTGAGCTCCAGGCCTTG.

Before inserting the HA tag into the endogenous genes, the plasmid was linearized with *NsiI* (NEB) followed by transfection with cytomix buffer (2 mM EDTA, 120 mM KCl, 0.15 mM CaCl_2_, 10 mM K_2_HPO_4_/KH_2_PO_4_, 25 mM HEPES, 5 mM MgCl_2_) into RH*∆hxgprt∆ku80* tachyzoites using a Nucleofector II electroporator (Amaxa Lonza, Basel, Switzerland) and one pulse with the T16 program. After 24 h, the positive transfectants were selected by adding 25 mg/mL of mycophenolic acid and 50 mg/mL xanthine before being cloned in a 96 well plate.

PCR using internal and external primers and Western blot analyses confirmed the insertion of the HA tag. The sequences of oligonucleotides used for confirmation were as follows: HA INT R, GCATAATCGGGCACATCATAG; KDAC4 EXT F, GCGCATCCTCATTCTGGA; TgKDAC4 F, **TACTTCCAATCCAATTTAATGC**TACGCTCATGGCCTAGGC (Figure 2B). The monoclonal rat anti-HA antibody (Clone 3F10-Roche, Mannhein Germany) was used for Western blot analyses at a dilution of 1:300, and the secondary anti-rat antibody conjugated with alkaline phosphatase (1:1000, Sigma, St. Louis, MO, USA).

### 2.3. Immunofluorescence Assays

Tachyzoite forms of TgKDAC4-HA and the control (untagged RH*∆hxgprt∆ku80*) were grown in confluent NHDF cells seeded on coverslips for 24 h. The intracellular parasites were fixed with 4% paraformaldehyde for 20 min at room temperature (RT). After being permeabilized with 0.2% Triton X-100 in PBS for 15 min and blocked with 1% albumin serum and 0.2% Triton X-100 in PBS for 30 min at RT, an incubation with monoclonal rat anti-HA was performed (Clone 3F10; Roche, 1:500, Mannhein, Germany). The samples were incubated with Alexa Fluor 488-conjugated anti-rat antibody diluted 1:600 (Molecular Probes, Invitrogen, Eugene, OR, USA). For colocalization assays with apicoplasts, samples were incubated with rabbit anti-HU antibodies diluted to 1:200 [38]. The latter antibody was detected using Alexa Fluor 546-conjugated anti-rabbit antibody diluted 1:600. All antibodies were diluted in blocking solution and incubated for 1 h at RT. Cells were incubated with 10 μM DAPI at RT for 10 min (4′,6-diamidino-2-phenylindole). Images were acquired using a Nikon Eclipse 80i microscope (Melville, NY, USA). For colocalization with the stromal apicoplast protein FNR, the TgKDAC4-HA tachyzoites were transiently transfected with the plasmid ptubFNRL-DsRed [44,45], and the cells were processed as described above. Images were acquired using a Leica DMI6000 B inverted fluorescence microscope (Wetzlar, Germany) (Magnification: 100×, NA: 1.4) with the accompanying deconvolution software (Leica LAS AF Lite. 3.1.0 build 8587).

### 2.4. Transmission Electron Microscopy

Tachyzoite forms of TgKDAC4-HA and the control (untagged RH*∆hxgprt∆ku80*) were inoculated on confluent NHDF cells and allowed to grow for 24 h. Infected host cells were washed with PBS 1X and fixed for 1 h at RT in a solution containing 0.1% glutaraldehyde and 4% paraformaldehyde in 0.1 M cacodylate buffer at pH 7.2. The samples were washed twice in 0.1 M cacodylate buffer (glutaraldehyde 25%, paraformaldehyde 10%, and cacodylate 0.1 M in distilled water). The parasites were dehydrated at −20 °C in increasing ethanol concentrations (30%, 50%, 70%, 90%, and twice in 100% ethanol). After gradual dehydration, the material was infiltrated in Lowicryl resin at −20 °C, under 100% ethanol resin (1:1) for 16 h. Subsequently, the material was included in pure resin at −20 °C, and the polymerization was carried out at −20 °C for 72 h under ultraviolet light.

Ultrathin sections (80 nm) were obtained with Leica C7 ultramicrotome and collected with 300 mesh nickel grids. The ultrathin sections were blocked with 3% BSA (Sigma-Aldrich, St. Louis, MO, USA) in PBS and labeled with rat anti-HA antibody (Roche, Mannhein Germany) for 1 h followed by 10 nm gold conjugated goat anti-rat IgG (Sigma-Aldrich, St. Louis, MO, USA). Sections were stained with 0.5% uranyl acetate in 2% methylcellulose and observed on a Fei TecNai G2 120 kV Spirit Electron Microscope at the Centro Nacional de Biologia Estrutural e Bioimagem (CENABIO)-INBEB, Rio de Janeiro, Brazil.

### 2.5. Immunoprecipitation of TgKDAC4 Complex

About 3 × 10^9^ intracellular parasites of untagged RH*∆hxgprt∆ku80* (control) and TgKDAC4-3HA were harvested from infected NHDF monolayers scraped from the 150 cm^2^ culture flasks. Infected cultures were then passed in a 26 G needle, resulting in a 0.2 g mass of tachyzoites. Parasites were washed with PBS 1X followed by an additional wash step including protease inhibitor (COMPLETE Mini Protease inhibitor cocktail tablet, Roche, Mannhein Germany). After centrifugation, the pellet was resuspended in the remaining PBS solution and then quickly frozen by dripping the precipitate directly into liquid nitrogen. The crosslinking step was unnecessary since the parasites were quickly frozen, and therefore the interactions were preserved as they were as closely as possible at the freezing time. The frozen cells were mechanically lysed by the Cryogrinding technique using the MM 400 vibrating grinder (Retsch, Haan, Germany), resulting in a powder [46].

The immunoprecipitation assay was performed with approximately 50 μg of the powder (equivalent to about 1 × 10^9^ parasites), resuspended in 1 mL of lysis buffer (50 mM Tris HCl pH 8, 150 mM NaCl, 1% NP40, 0.5% sodium deoxycholate, 0.1% SDS, 2 mM EDTA), followed by sonication at potency 5, with ten repetitions for 10-s pulses in an Ultrasonic Homogenizer 4710 Series sonicator (Cole-Parmer Scientific Experts, Vernon Hills, IL, USA). The sample was centrifuged at 20,000× *g* for 10 min at 4 °C. The supernatant was incubated with 50 μL (1.5 mg) magnetic beads coupled to protein A (Invitrogen) with 0.5 μg anti-HA (Roche, Mannhein, Germany) for 16 h in lysis buffer at 4 °C. The samples were incubated for 6 h at 4 °C followed by six washing steps by inversion, with washing buffer (50 mM Tris HCl pH 8, 150 mM NaCl, 1% NP40, 0.5% sodium deoxycholate, and 2 mM EDTA). Samples containing TgKDAC4-3HA and associated proteins as well as the control were eluted with 1% SDS, 50 mM Tris HCl pH 8, and 10 mM EDTA [46,47].

### 2.6. Mass Spectrometry and Data Analysis

After separating the proteins eluted by SDS-PAGE, each gel lane was excised out of the gel and each fraction was cut into 1 × 1 mm pieces. After destaining, proteins were reduced with 10 mM DTT at 56 °C for 1 h and alkylated with 55 mM iodoacetamide at 25 °C for 45 min, protected from light. Subsequently, gel pieces were incubated with 12.5 ng/μL trypsin (Promega, Madison, WI, USA) in 50 mM ammonium bicarbonate at 37 °C for 18 h for protein digestion. Then, peptides were extracted from the gel matrix, concentrated by vacuum centrifugation, and purified using homemade RP-C18 StageTip columns prior to MS analysis. The peptides were analyzed by LC-MS/MS in triplicate in an Ultimate 3000 RSLCnano coupled to an LTQ Orbitrap XL ETD mass spectrometer (Thermo Scientific, Waltham, MA, USA) (Mass Spectrometry Facility RPT02H/Carlos Chagas Institute-Fiocruz, Curitiba, Paraná, Brazil) equipped with a nanoelectrospray ion source (Thermo Scientific, Waltham, MA, USA). Peptide samples were fractionated via chromatographic separation in a 15 cm fused silica emitter (75 µm inner diameter) packed in-house with reversed-phase ReproSil-Pur C18-AQ 3 µm resin (Dr. Maisch GmbH, Ammerbuch-Entringen, Germany). Chromatography was carried out at 250 nL/min with a 5 to 40% acetonitrile gradient in 0.1% formic acid in a 60 min gradient. The parameters used were as follows: 2.7 kV spray voltage, 100 μA spray current, 35 V capillary voltage, 100 V tube lens, and 175 °C tube transfer. Full-scan mass spectra were acquired in the orbitrap within an *m/z* window ranging from 300 to 2000, resolution of 60,000 at 400 *m*/*z*, and AGC target of 106. The option lock mass was enabled at 401.922718 *m*/*z* to improve mass accuracy. MS/MS was carried out in the linear ion trap, where the ten most intense precursor ions from each full scan were isolated at an AGC target value of 3 × 10^4^ and fragmented by CID and detected in the linear trap quadrupole (LTQ). Protein validation, quantification, and identification were performed in the MaxQuant platform (version 1.6.3.4) set to default parameters. Former target ions selected for MS/MS were dynamically excluded for 90 s. Proteomic data analysis was performed in Perseus (version 1.6.14.0, Max Planck Institute of Biochemistry, Planegg, Germany) as previously described [48], with minor changes. The MaxQuant output data were initially filtered by removing contaminants and hitting the reverse database, with proteins identified only by a modification site. The label-free quantification (LFQ) intensities were log2-transformed. The identified proteins were filtered for at least two valid LFQ intensity values in at least one replicate group. LFQ intensity correlation multi-scatterplots were used to evaluate reproducibility among replicates, showing a Pearson correlation coefficient for each comparison. Before the statistical analysis, missing LFQ-Intensity values were imputed from normal distribution around the detection limit (width set at 0.3, downshift of 2.0 standard deviations). A two-tailed Student’s *t*-test (S0 = 0, threshold *p*-value = 0.05) was performed.

### 2.7. Phylogenetic Analysis

A Hidden Markov profile was created based on the alignment of four KDAC4 sequences from apicomplexan parasites (*Toxoplasma* strains ME49, VEG and GT1, and *Hammondia*) in Hmmer v3.2.1. The profile was used to mine data from the reference proteome database of UniProt in the EBI Hmmer web server. We discarded sequences that did not have the histone deacetylase domain according to the Pfam database (PF00850), entries with E-values higher than 9.99 × 10^−10^, and sequences with fewer than 300 amino acids. The remaining sequences were analyzed using the Web Batch CD-Search tool against NCBI’s conserved domain database (CDD) and trimmed to keep only the histone deacetylase domain region. Sequences found with partial domains were removed, resulting in 7402 sequences. They were aligned using MAFFT 7.402, and the resulting alignment was trimmed to remove positions with more than 95% gaps and less than 0.05 similarity score using TrimAl 1.2.59. A phylogenetic tree was inferred using ML and 1000 ultrafast bootstrap replicates using IQ-TREE 1.6.10. The tree was visualized in IToL.

## 3. Results

### 3.1. TgKDAC4 Has a Putative Prokaryotic Origin

In silico assays using the *Toxoplasma* database (ToxoDB: https://toxodb.org/toxo/app; accessed on 20 March 2015) made it possible to identify a partial sequence of the gene coding for TgKDAC4 (TGME49_257790). The complete sequence of *TgKDAC4* was then obtained by amplifying cDNA followed by sequencing (Figure S1). We observed that the obtained sequence was a coding region of 3495 bp composed of six exons rather than four as annotated in the database, corresponding to a protein of 123 kDa. The obtained sequence agrees with the previous analysis by Ramaprasad and col., who performed a manual analysis of the *T. gondii* genome [49] and confirmed the presence of two more exons in the TgKDAC4 gene (A. Ramaprasad, personal communication, 3 March 2016).

Searches in the Conserved Domain Database (CDSEARCH/cdd) confirmed the presence of a complete KDAC domain of class IV at the C-terminal region of TgKDAC4. The sequence similarity analysis showed that the KDAC domain is highly similar to domains of lysine deacetylases from bacteria, suggesting a prokaryotic origin. Interestingly, the constraint-based Multiple Alignment Tool from NCBI demonstrated that the protein is specific to the Apicomplexa phylum and similar to enzymes from *Neospora caninum*, *Besnoitia besnoiti*, and *Cystoisospora suis* (Figure S2).

**Figure 1:**
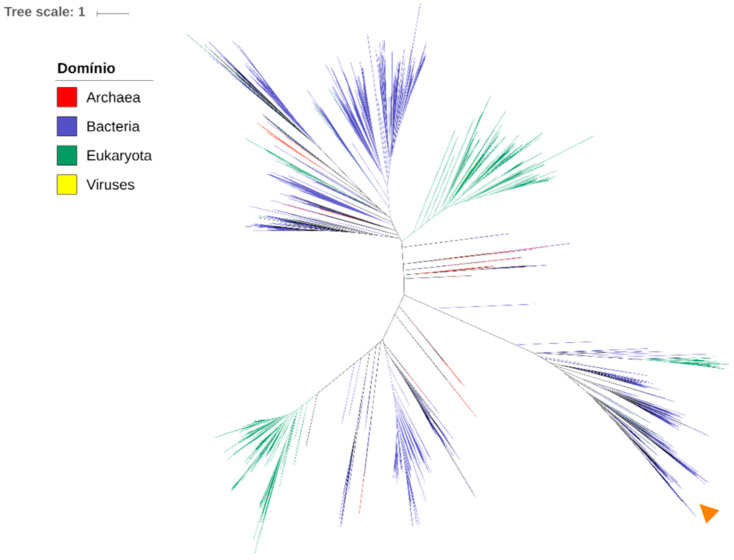
Phylogenetic tree constructed from 7395 histone deacetylase domain sequences. The inner colors represent the taxonomic domains (Eukarya, Bacteria, Archaea, and viruses). The orange arrow indicates the position of KDAC4 from the three different strains of *Toxoplasma* (ME49, GT1, and VEG) and *Hammondia* KDAC4.

To assess whether TgKDAC4 has a prokaryotic origin, we performed a phylogenetic analysis including all deacetylases with domains similar to TgKDAC4, including TgKDAC4 from three different strains of *Toxoplasma* (ME49, GT1, and VEG) and *Hammondia hammondi* (HHA_257790), which has a high identity with TgKDAC4 (83%). KDAC enzymes of Archaea (170 sequences), Bacteria (3820 sequences), Eukarya (3402 sequences), and viruses (3 sequences) were also included in the phylogenetic analyses. The resulting phylogenetic tree showed TgKDAC4 grouped into a branch formed by proteins of bacterial origin. This result suggests that TgKDAC4 is more similar to bacterial than eukaryotic proteins, which may indicate a bacterial origin (Figure 1).

### 3.2. TgKDAC4 Localizes in the Apicoplast

**Figure 2:**
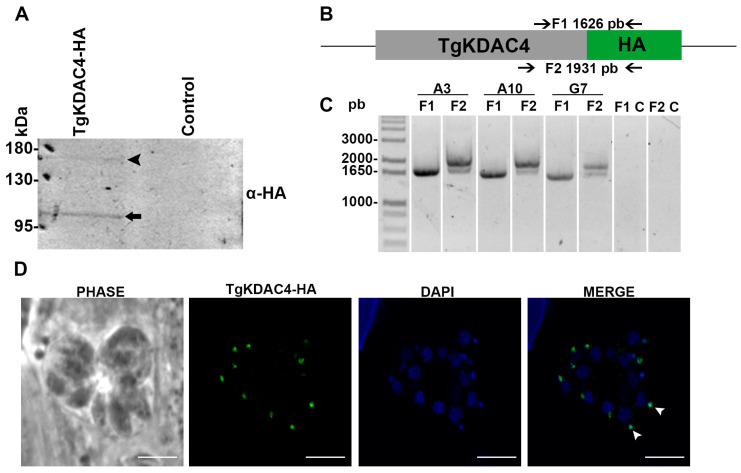
TgKDAC4-HA inside the apicoplast of *T. gondii*. (**A**) Western blot analysis confirmed HA-tag on TgKDAC4 protein. The bands corresponding to the mature (black arrow) and immature (black arrowhead) forms of TgKDAC4 are shown. Primary antibody: anti-HA. Secondary antibody: anti-mouse conjugated to alkaline phosphatase. (**B**) Design of internal and external primers to confirm the tag sequence 3XHA in the C-terminal portion of *tgkdac4* by PCR amplification. (**C**) The PCR product was visualized on 1% agarose gel, as shown for three clones (A3, A10, and G7). Ladder: 1 KB Plus (Invitrogen). C: negative control. (**D**) Immunolocalization of TgKDAC4 in intracellular tachyzoite forms of *T. gondii* (Magnification: 100×, NA 1.4). An indirect immunofluorescence assay was performed against the HA tag added to the C-terminal portion of endogenous TgKDAC4 protein. The white arrowheads indicates the apicoplast. The nucleus and the apicoplast were stained with DAPI (blue).

**Figure 3:**
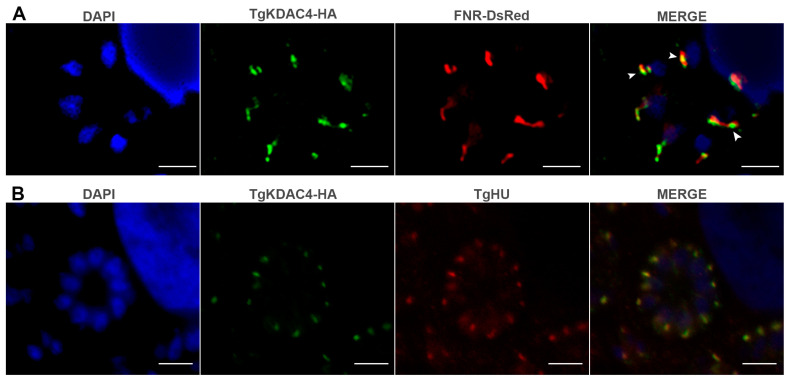
TgKDAC4-HA inside the apicoplast of *T. gondii*. (**A**) Immunolocalization of TgKDAC4 with apicoplast after transfection with FNR-DsRed. The arrowheads indicate the apicoplast (The vector was constructed by [45]. (**B**) Immunolocalization of TgKDAC4 and TgHU inside the apicoplast. The nucleus and the apicoplast were stained with DAPI (blue). All images are at the same magnification (100×, NA: 1.4).

**Figure 4:**
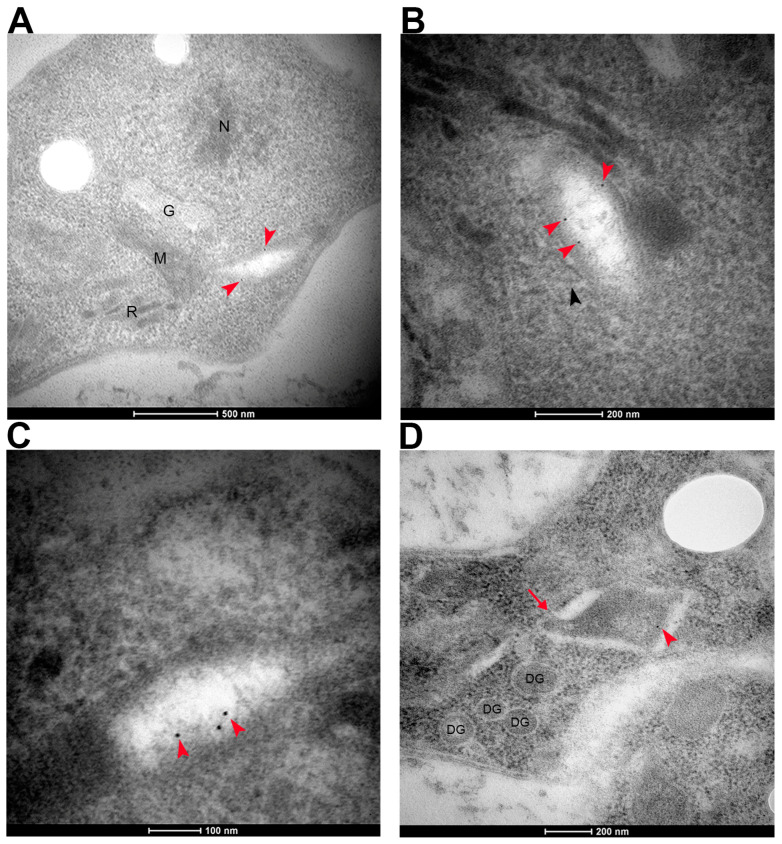
Immunostaining of TgKDAC4 in tachyzoite forms of *T. gondii*. Immunostaining was performed using anti-HA (clone 3F10-Roche, Mannhein Germany) to label the endogenous TgKDAC4-HA. Red arrows indicate the labeling of TgKDAC4. The black arrow indicates the endoplasmic reticulum. The red arrow indicates the membranes region. N: nucleus, G: Golgi complex, M: mitochondria, R: rhoptries, DG: dense granules. Bars: from 100 nm to 500 nm, as indicated. (**A**–**D**) show four electron microscopy sections labeled for TgKDAC4 (arrowheads).

The apicoplast is an organelle unique to the phylum Apicomplexa. It has been suggested that this organelle has its origin in an assimilation event similar to mitochondria and chloroplasts, in which a primitive prokaryotic organism was incorporated into a eukaryotic one [35]. Since apicoplast proteins are similar to bacterial proteins, we hypothesize that TgKDAC4 may have been exported to this organelle. Furthermore, we predicted the subcellular location of the protein in this organelle to a probability of 61% using Deeploc (DeepLoc-1.0 server) (Figure S3). We constructed an HA-endogenous tagged line (TgKDAC4-HA) to confirm this prediction. Experimental approaches using the **TgKDAC4-HA** parasites detected a protein of around 100 kDa (arrow in Figure 2A). Although the previous prediction described a protein of 124 kDa, we confirmed the correct insertion of a 3X-HA sequence in the endogenous gene (Figure 2B,C), which is likely due to the processing in the N-terminal region, a common feature of apicoplast proteins. Fluorescence microscopy analysis using an anti-HA antibody made it possible to observe TgKDAC4-HA colocalizing with the apicoplast genome (arrowheads in Figure 2D and Supplementary Video S1). This same analysis, with deconvolution processing in a parasite strain expressing fluorescence for the apicoplast protein ferredoxin-NADP+ reductase (FNR)-DsRed, also confirmed the presence of TgKDAC4 in the apicoplast. Hence, this antibody labeling showed TgKDAC4 colocalizing with the protein FNR-DsRed, located in the apicoplast stroma (arrows in Figure 3A). However, we noted an incomplete overlap between TgKDCA4 and the stromal protein TgFNR, suggesting that TgKDAC4 may be located at the periphery of the organelle. Reiff et al. (2012) reported and characterized a prokaryote histone-like protein (HU) located in the apicoplast of *T. gondii*. Thus, TgHU colocalizes with the apicoplast genome and may be responsible for genome compaction [38]. Considering the role of KDACs in modifying histones, we assessed a possible colocalization of TgKDAC4 and TgHU. Hence, we labeled tachyzoites of *T. gondii* with anti-HA and anti-HU antibodies. Fluorescence microscopy analysis showed that TgKDAC4 and TgHU have a similar localization pattern (Figure 3B), suggesting a possible interaction between both proteins. Electron microscopy confirmed the presence of TgKDAC4 inside the apicoplast, as the protein was constantly found in a compartment with regular topology closely located to the nucleus and Golgi complex (red arrowheads in Figure 4A). It is also possible to visualize the contact between the apicoplast and the endoplasmic reticulum membrane (black arrowhead in Figure 4B) and a region with the apicoplast membranes (red arrow in Figure 4D).

### 3.3. TgKDAC4 Interacts with Apicoplast Proteins

To further understand the function of this apicoplast lysine deacetylase, we searched for TgKDAC4 interactors using immunoprecipitation (IP) followed by mass spectrometry (MS) identification (IP-MS).

Triplicate tachyzoite extracts from both TgKDAC4-3HA and untagged control parasites were prepared using the cryogrinding method [46], and KDAC4-3HA was immunoprecipitated using magnetic beads coupled to protein A associated with anti-HA.

**Figure 5:**
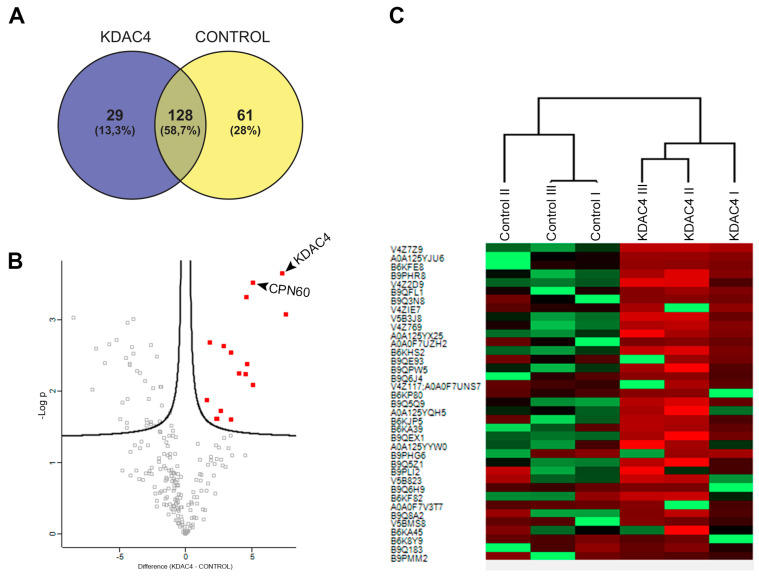
Identification of TgKDAC4 interactors by IP-MS. (**A**) Venn diagram of proteins exclusively identified in TgKDAC4 HA extracts compared to control WT cell lines. (**B**) Volcano plot highlighting proteins statistically enriched in TgKDAC4 IP extracts. The *x*-axis represents the abundance (intensity) of proteins in each IP (TgKDAC4 versus CONTROL). The *y*-axis represents the −log *p*-value of the *t*-test, FDR 1%. (**C**) Hierarchical clustering of exclusively and statistically enriched proteins in tgKDAC4 extracts compared to control. Red indicates enriched protein; green indicates reduced levels of the protein.

Pearson and PCA analysis of MS data from the replicates indicated a high correlation between them (Figure S4). A total of 218 proteins were detected, which included 29 proteins exclusively detected at IP extracts of TgKDAC4 cells (Figure 5A and Table S1), including TgKDAC4 itself. When we refer to exclusivity, we include the proteins that did not appear in control samples, and therefore the statistical differences could not be calculated. Among them, CPN60 (TGVEG 240600) [50], GAPDH2 (TGVEG_269190) [51], and ribosomal protein RPL14 (TGVEG_267060) [52] are located in the apicoplast. Acetylation sites were identified for CPN60 and GAPDH2, representing possible targets for TgKDAC4 [10]. We further evaluated proteins that were statistically enriched in TgKDAC4 extracts. In that case, the proteins were identified in both TgKDAC4 extracts and control samples. The volcano plot indicated an enrichment of 14 proteins (Figure 5B and Table 1), with CPN60 five times enriched in IP extracts. The interaction between TgKDAC4 and TgCPN60 was confirmed by western blot (Figure S4). The hierarchical clustering shows exclusively and statistically enriched proteins in TgKDAC4 extracts compared to control parasites (Figure 5C).

We also identified nuclear, cytoplasmatic, mitochondrial, Golgi apparatus, and endoplasmatic reticulum proteins that may interact with TgKDAC4 during its transport to the plastid. Table S2 shows all protein partners identified in MS analysis.

## 4. Discussion

*Toxoplasma* has seven lysine deacetylases, of which five have a classical KDAC domain, and two belong to the sirtuin class. In 2012, the KDACs from *T. gondii* were classified into four classes according to homology with yeast KDACs, except for TgKDAC4, which, according to pfam, is grouped in class IV [20]. Class IV is the least understood class of deacetylases, with KDAC11 from humans being the best characterized [19,20,23,24,25]. KDAC11 is found in the nucleus [53] and interacts with the promoter region to regulate the expression of interleukin-10 (IL-10) in antigen-presenting cells negatively [54]. HDAC6 (Class II) also interacts with the promoter region of the IL-10 gene and is a transcriptional activator protein, representing the first example of two HDACs with opposite functions acting on the same promoter [55].

Lysine deacetylases are known mainly for acting in deacetylating histones that lead to gene transcription control or serve as signalers for cellular processes. However, KDACs can also deacetylate several other proteins, increasing the range of possible functions in critical cellular processes. Recently, the deacylation of lysines was also associated with KDACs [56]. Human KDAC11 is capable of deacylating fatty acids, effectively removing decanoyl, dodecanoyl, and myristoyl groups [21].

The present study focuses on TgKDAC4, a class IV deacetylase, with a domain similar to bacteria lysine deacetylases (Figure S2). To better understand the evolution of TgKDAC4, we performed a phylogenetic analysis of KDAC4 of *Toxoplasma* and *Hammondia* and detected a possible bacterial origin for this enzyme (Figure 1). Furthermore, TgKDAC4 is located in the *T. gondii* apicoplast, a plastid-like organelle (Figure 2, Figure 3 and Figure 4). Furthermore, the prokaryotic acetoin utilization protein (AcuC) may have been the origin of eukaryotic class I KDACs, while the acetyl polyamine amidohydrolase protein (Apah) may have been the origin of class II. Although human KDAC11 is a deacetylase of class IV, it appears to be related to the prokaryotic AcuC protein, with similarities in the sequence of the active site [57,58,59]. In addition, KDACs of class IV share a close phylogenetic relationship with eubacterial proteins. However, the search for proteins similar to human KDAC11 did not uncover any class IV KDAC in apicomplexan parasites [60]. Interestingly, the phylogeny data revealed the appearance of a virus lysine deacetylase (from *Tupanvirus*). *Tupanvirus* has a giant genome and infects protists, including apicomplexan parasites [61].

Most apicoplast proteins are encoded in the nucleus and transported to the plastid after translation through a bipartite sequence in their N-terminal end, composed of a classic signal peptide and a plant-like transit peptide [47,62,63]. This signal peptide directs the protein to the endoplasmic reticulum, while the transit peptide is essential for the transport of the protein into the apicoplast. Although the signal peptide is conserved, the transit peptide is not, meaning that a consensus motive cannot be identified [40,64]. These signals are cleaved after the protein enters the apicoplast, causing the well-known two bands that apicoplasts proteins tend to show in Western blot assays, denoting an immature protein before cleavage and the mature protein after the removal of the signal sequence. The TgKDAC4 protein showed two bands in the Western blot (Figure 2A), indicating the processing of the protein after the signal peptide cleavage [47,62,63]. Apicoplast localization was then confirmed by immunofluorescence and electron microscopy (Figure 3 and Figure 4). 

Although TgKDAC4 localization was unexpected, it is not unprecedented; OsHDAC6, a lysine deacetylase, has already been identified in the chloroplast of rice (*Oryza sativa*) [59]. Interestingly, OsHDAC6, whose function is not yet elucidated, has also been described as a member of class IV [59]. In *Arabidopsis thaliana*, a class II deacetylase (HDA14), dual-localization in the chloroplast and mitochondria were also identified, and most of its targets have a role in photosynthesis [65]. It is worth mentioning that, although apicoplast is a plastid-like organelle, it is non-photosynthetic [35].

Acetylated proteins are involved in several cellular functions and have different locations, including in specific organelles of apicomplexan parasites, such as the apicoplast. A previous study identified acetylated proteins of *T. gondii* using mass spectrometry and described acetylated residues in known proteins, such as histones and tubulin, as well as 411 new acetylation sites in 274 tachyzoite proteins [10]. To understand the function of TgKDAC4 in the apicoplast, we perform an immunoprecipitation assay to identify targets or partners. Our results identified proteins with described acetylation sites. Among those, two known apicoplast proteins (TgCPN60 and TgGAPDH2) were identified.

*Toxoplasma* has two GAPDHs: GAPDH1, which is a cytoplasmatic protein, and GAPDH2, located in the apicoplast [31,51]. GAPDH is considered a multifunctional protein in humans that plays an essential role in glycolysis and other processes, such as heme metabolism and intracellular membrane trafficking [66,67,68]. Acetylation in GAPDH can increase activity, change the protein location, and initiate a cell death cascade, depending on the acetylated lysine [3,66,69].

TgCPN60 is a protein located in the stroma of the apicoplast that has the function of protein maturation [50,70]. In addition, acetylome analysis of *Plasmodium* identified the acetylation sites of CPN60 [71]. CPN60, also known as GroEL, has several acetylation sites in bacteria. However, the function of these modifications remains unclear [72].

Several complexes with histone deacetylases of different biological functions have been described. In mammals, the deacetylation complex (NuRD-nucleosome remodeling deacetylase) comprises helicases, chaperones, proteins with zinc finger domains, and proteins with CpG binding. Except for the TgCPN60 chaperone, no other protein with these functions was found in the immunoprecipitation of TgKDAC4 in the present study, suggesting that it has a different deacetylation mechanism [73,74]. However, we cannot compare the complexity and compaction of the DNA contained in the nucleus to the DNA of the apicoplast, where the only histone-like protein found was TgHU [38]. In *T. gondii*, the only lysine deacetylase whose complex has been purified was TgKDAC3, a nuclear protein. This analysis showed the presence of a corepressor complex known as TgCRC. TgCRC is composed of the proteins CRC, actin, transducin beta-like protein 1 (TgTBL1), TCP1 ring complex, and chaperones [12].

Using immunolocalization assays, we were able to verify that TgHU and TgKDAC4 are located in the same region of the apicoplast (Figure 3B), suggesting that TgKDAC4 may be related to the nucleoid of this organelle. The genome of the apicoplast comprised 25 copies of circular DNA, and the histone-like protein TgHU is important for apicoplast DNA compaction, although the complete function remains unclear [38,75]. However, although the HU of *Mycobacterium tuberculosis* acetylates in different lysines, the same seems not to occur in *Toxoplasma* TgHU, since it was not detected in our study or in studies of the acetylome [10,38,76].

Treatment of apicomplexan parasites such as *Toxoplasma* and *Plasmodium* is associated with resistance and severe side effects, highlighting the importance of the search for new drugs. Lysine deacetylases can affect gene expression and protein activity and are considered potential drug development targets, including for cancer treatment. In 1996, Darkin-Rattray and colleagues discovered apicidin, a cyclic tetrapeptide isolated from *Fusarium* ssp., with an inhibitory effect against *Plasmodium* KDACs [77,78,79]. Another cyclic tetrapeptide studied is FR235222, which was isolated from the fermentation broth of *Acremonium* species. This drug explicitly inhibits TgKDAC3, decreasing the intracellular growth of *T. gondii* and inducing differentiation from tachyzoites to bradyzoites [26,80]. Therefore, KDACs are promising drug targets for toxoplasmosis treatment.

As explained above, in addition to being an important gene target, the localization of TgKDAC4 at the apicoplast can confer an additional advantage for drug development. The apicoplast is essential for the parasite’s survival; therefore, it is considered a promising target for treating apicomplexan-related diseases. Due to its origin, it is not surprising that the apicoplast genome is very similar to that of prokaryotes, showing similar replication, transcription, and translation machinery components. Some described antibacterial drugs targeting this machinery have been shown to kill these parasites [81,82,83]. As a result, some scientists consider the apicoplast the “Achilles’ heel” of these parasites [35].

In conclusion, the present study revealed that TgKDAC4 is an enzyme unique to some apicomplexan parasites that are located in the apicoplast. Although its function and importance in this essential organelle need further investigation, the search for new chemotherapy targets stimulates the study on this enzyme that seems promising as a new target for the development of drugs for toxoplasmosis treatment.

## Figures and Tables

**Table 1:** Protein complex identified by mass spectrometry. Proteins statistically enriched in TgKDAC4 IP extracts.

ID	Protein Name	Difference (KDAC4-WT)	(−LOG (*p*-Value))
V4Z7Z9	Uncharacterized protein (KDAC4 domain Fragment)	7.557988803	3.07964558
A0A125YJU6	Histone deacetylase KDAC4	7.262962341	3.654127752
B6KFE8	Putative chaperonin cpn60	5.038188934	3.522729209
B9PHR8	Protein phosphatase 2C domain-containing protein	5.025075277	2.092972874
V4Z2D9	Uncharacterized protein (Fragment)	4.627913793	2.386059714
B9QFL1	Putative transmembrane protein	4.563500086	3.319557656
V4ZIE7	Uncharacterized protein	4.480522792	2.244625048
V5B3J8	Putative transmembrane protein	4.005473455	2.253793632
V4Z769	Kelch motif domain-containing protein	3.407096227	1.610467411
A0A125YX25	Microneme protein putative	3.376639048	2.540889206
B6KHS2	Dense granule protein GRA9	2.832564672	2.637648916
B9QPW5	Proline-rich protein	2.602699916	1.727458536
B6KP80	Uncharacterized protein	2.340368907	1.618390219
B9Q5Q9	Putative transmembrane protein	2.286419551	1.619608828
B9QEX1	SAG-related sequence SRS44	1.815659205	2.686913967
B9Q5Z1	Cyst matrix protein	1.570529302	1.878802921

## Supplementary Materials

The following supporting information can be downloaded at: https://www.mdpi.com/article/10.3390/microorganisms11061558/s1, Figure S1: Amino acid sequence of KDAC4. The complete sequence of TgKDAC4 was then obtained by amplifying cDNA followed by sequencing. Figure S2: Alignment of TgKDAC4. The alignment shows that TgKDAC4 (Query, first line) is specific to the Apicomplexa phylum and similar to enzymes from *Neospora caninum*, *Besnoitia besnoiti*, and *Cystoisospora suis*. KDAC domain shows high similarity with domains of lysine deacetylases from bacteria, suggesting a prokaryotic origin (provided by NCBI Multiple Sequence Alignment). Figure S3: Prediction of the location of TgKDAC4. The prediction of the subcellular location of TgKDAC4, provided by DeepLoc Server, shows a possible signal in the N-terminal portion to lead the protein transport to the apicoplast. Figure S4: Confirmation of the interaction between TgKDAC4 and TgCPN60. Western blot of immunoprecipitation eluate confirming the interaction of TgKDAC4 (red) and TgCPN60 (green). Western Blot using primary anti-HA antibody and CPN60 antibody. About 1 × 109 intracellular parasites were used. Marker: PageRuler Prestained Protein Ladder. Figure S5: Data quality and reproducibility analysis. (A) Scatter plot between biological replicates of IP extracts of TgKDAC4 (KDAC4 I–KDAC III) and wild-type cells (Control I-Control III). Pearson correlations are depicted above each scatter plot. (B) PCA plot analysis. Table S1: Proteins found exclusively in IP extracts of TgKDAC4 cells. 29 proteins exclusively detected in IP extracts of TgKDAC4 cells. Among them, TgKDAC4 itself (TGVEG_257790/TGVEG_441840), CPN60 (TGVEG 240600), GAPDH2 (TGVEG_269190), and ribosomal protein RPL14 (TGVEG_267060), all located in apicoplast. Table S2: All protein partners identified in MS analysis. Video S1: Serial imaging and 3D reconstruction of TgKDAC4 (green) in the apicoplast of intracellular tachyzoite forms of *T. gondii*. The nucleus and the apicoplast were stained with DAPI (blue).
