# Supplementary material for: TgKDAC4: A Unique Deacetylase of Toxoplasma’s Apicoplast
Source: Microorganisms. 2023 Jun 12;11(6):1558. doi: 10.3390/microorganisms11061558 (PMC10302316; doi:10.3390/microorganisms11061558)
Supplement: Supplementary file 1 [file microorganisms-11-01558-s001.zip › Supplementary figures_May.pdf]

6. SUPPLEMENTARY FIGURES

>KDAC4
M G H P P P W T A S P L F F L L L F W H A P P S L V S S C L H A G H A L H V H
P A H N P S L P P L S L P S D R P G A A G D G Q T G A S L V H N A R A A L P L
C A S R V F A G G A L Q P S T A V P P R T Q P L H R C A R R Y A P S G R L L L
K P R L G L K A L P I H E V A E G R A E Q E D R A R P P D P S V S G R T L T C
V T L R T K E T C N D A D C G G L A C R T G N A T I A G R G A R A S D G R Q P
P T K Q E G V F P V A T S F R F S S R S L R S S T P L S R Y F L S S S P S E N
K F E F H H F L S Y T V T A E L P T S A F T F V D A P Q S L T S C A D V W S H
P C R A F V F F P L Q R S R T S V G F S P S S H S I R P S P R R W F S S V N L
C A L R A S F G S I A S S C S S P V G P D H S S L T A V A I T R A V R C F P S H
S A P F P P S S S C D L L S A S S P S H A S L P A C S A S A S S S S P P S R A
R P T V C L Y V P R T H S P R P P R S V G S A A S P S S G G P R V S Q S E G
T P S G P V D S P L S P T S R G S C S Y S T P S V S C P G G L L L S L P S V S
P V S L S T S L R H F L V S Y T S P F P A R S L P L I F V D A G L S L A S D A S
L H A A R P V G L S Q E H R E T C A S A L G S L I D T A V A S N A V A Q T S C
E C R P R G L P I V Y S P R L V P P S F P R N H C L Q P Q K L G R L F S F L T H
P S G A S N L R T P E S G V T K T W E A R E A E I A K A T S G V E G S H E F R
W D Y V T V H S D L R P K R L N S D H P V T K A G I G V G E N R G R E K N G
E D G R P S E A G C E K S T S P S H A L S P S A S V P H G P V D A T R Q N G
C G R D D P P R C T H P A G V Q V T A G N E L N R N P L L A G S H D Q H M D
S T R A T L G T S V S S C F S L F S P I H D A D V T R A W L Q V V H A P E Y V
C A A S A A V L S E E E E R K I G F P V T K G Y A D K S L A E V S S T V L G T
W L A F H F G L A C V V G G G T H H A K T D S G G K F C V F N D V A V A A A
L A L K Q G I A E R I L I L D L D V H Q G D G T A E I F S N E P R V K T V S I H C
E D N F P F P K A Q S D V D I G L P A G T G D E V Y L R Q L N E V L P R V L L
E H R P T L I L Y V A G V D I H E Q D T F G N F Q I T D A G L R M R E E L V F S
H C L R Y N Q A I L R H R R Q I E A R L R S P D A E E K Q A K H P R E P R T A
E G Q T S N S S G S Q T S S P E D L F S R S P G R S Q T W E D L G E R E V P
V E Q R V G V S G H E V Q G N A G S L P D R K E S R D A R A V H A E T L K T
V T H A D T C H P V A I C S V V A G G Y S D D I A H T V R R H A I L F Q T A A R
F W K D R R Y P E F Y R P P Y K A W S S E

Supplementary Figure S 1: Amino acid sequence of KDAC4. The complete sequence of *TgKDAC4* was then obtained by amplifying cDNA followed by sequencing.

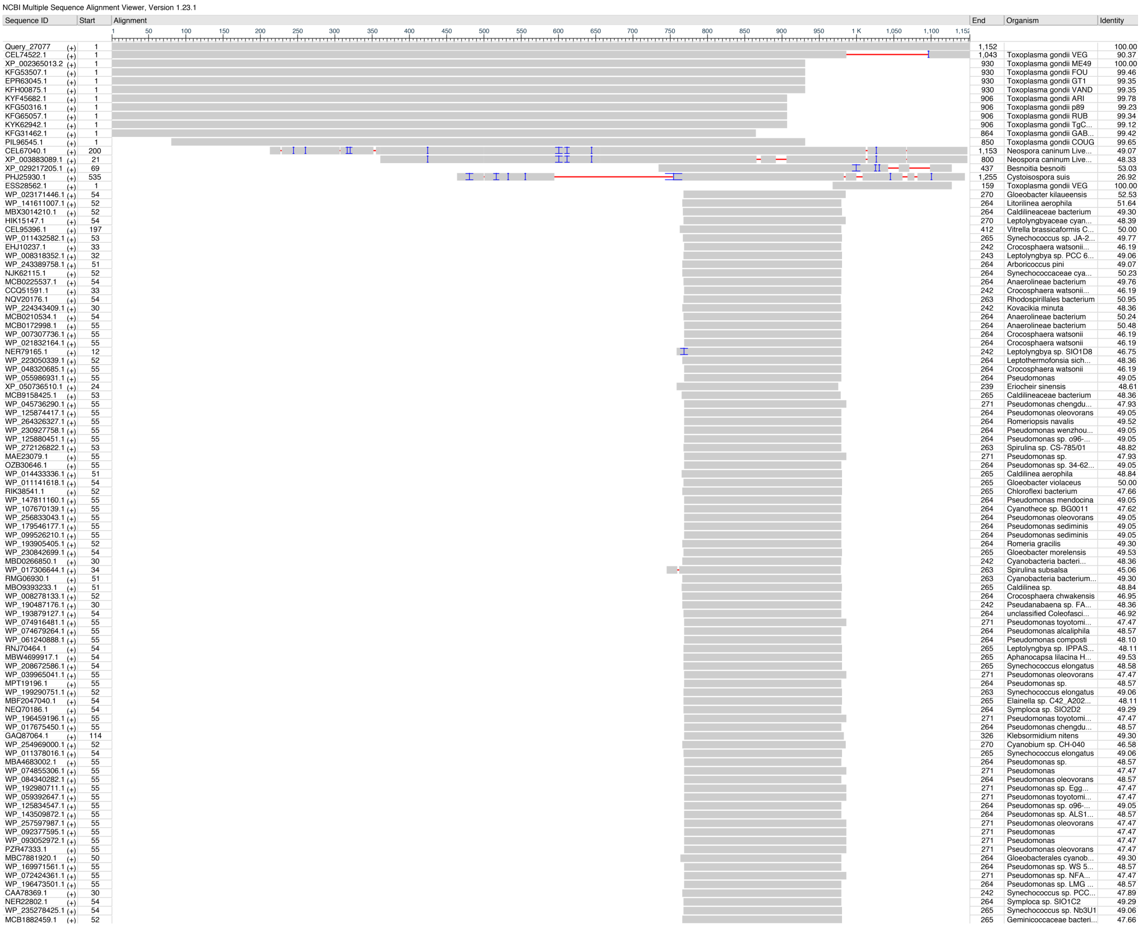

Supplementary Figure S 2: Alignment of TgKDAC4. The alignment shows that TgKDAC4 (Query, first line) is specific to the Apicomplexa phylum and similar to enzymes from *Neospora caninum*, *Besnoitia besnoiti*, and *Cystoisospora suis*. KDAC domain shows high similarity with domains of lysine deacetylases from bacteria, suggesting a prokaryotic origin (provided by NCBI Multiple Sequence Alignment).

Localization	Plastid	Extracellular	Nucleus	Cytoplasm	Endoplasmic reticulum	Mitochondrion	Lysosome/Vacuole	Cell membrane	Golgi apparatus	Peroxisome
Likelihood	0.619	0.09	0.0608	0.0554	0.0462	0.0416	0.0343	0.0231	0.0166	0.0129

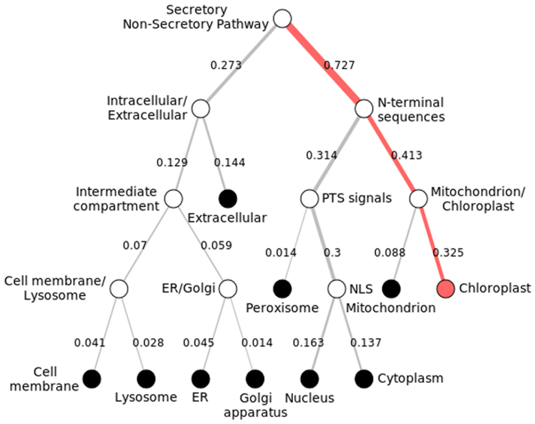

Supplementary figure S3: Prediction of the location of TgKDAC4. The prediction of the subcellular location of TgKDAC4, provided by DeepLoc Server, shows a possible signal in the N-terminal portion to lead the protein transport to the apicoplast.

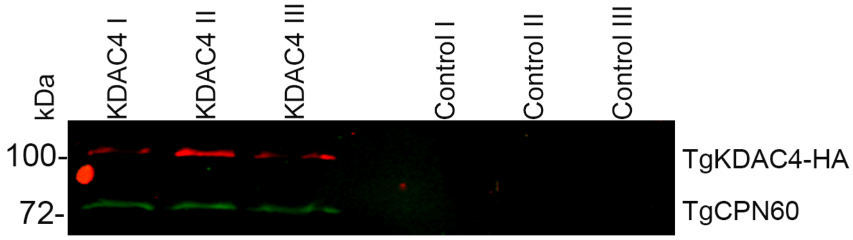

Supplementary Figure S4: Confirmation of the interaction between TgKDAC4 and TgCPN60. Western blot of immunoprecipitation eluate confirming the interaction of TgKDAC4 (red) and TgCPN60 (green). Western Blot using primary anti-HA antibody and CPN60 antibody. About 1 x 10^9 intracellular parasites were used. Marker: PageRuler Prestained Protein Ladder.

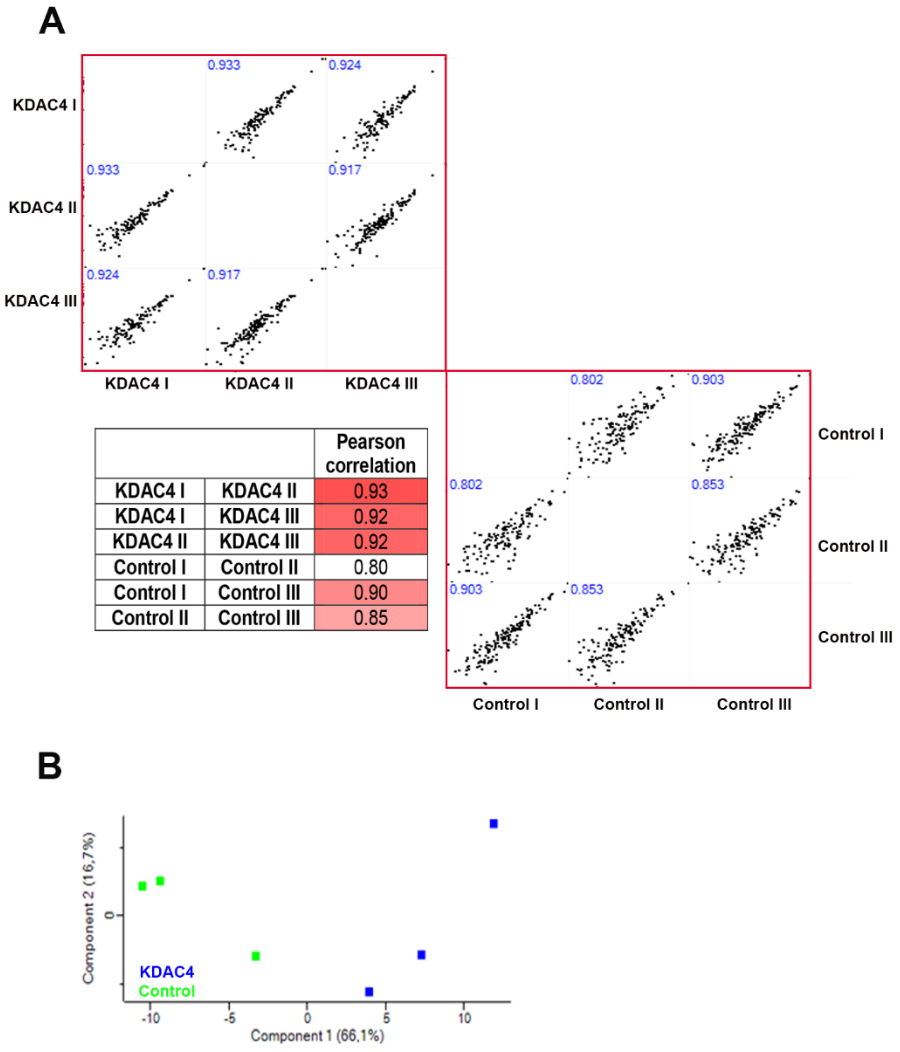

		Pearson correlation
KDAC4 I	KDAC4 II	0.93
KDAC4 I	KDAC4 III	0.92
KDAC4 II	KDAC4 III	0.92
Control I	Control II	0.80
Control I	Control III	0.90
Control II	Control III	0.85

Supplementary Figure S5: Data quality and reproducibility analysis. (A) Scatter plot between biological replicates of IP extracts of TgKDAC4 (KDAC4 I–KDAC III) and wild-type cells (Control I-Control III). Pearson correlations are depicted above each scatter plot. (B) PCA plot analysis.

Supplementary table S1: Proteins found exclusively in IP extracts of TgKDAC4 cells.

29 proteins exclusively detected in IP extracts of TgKDAC4 cells. Among them, TgKDAC4 itself (TGVEG_257790/TGVEG_441840), CPN60 (TGVEG 240600), GAPDH2 (TGVEG_269190), and ribosomal protein RPL14 (TGVEG_267060), all located in apicoplast.

Supplementary table S2: All protein partners identified in MS analysis.

Supplementary video S1: Serial imaging and 3D reconstruction of TgKDAC4 (green) in the apicoplast of intracellular tachyzoite forms of *T. gondii.* The nucleus and the apicoplast were stained with DAPI (blue).
